# Study on Synergistic Mechanism of Inhibitor Mixture Based on Electron Transfer Behavior

**DOI:** 10.1038/srep33252

**Published:** 2016-09-27

**Authors:** Peng Han, Yang He, Changfeng Chen, Haobo Yu, Feng Liu, Hong Yang, Yue Ma, Yanjun Zheng

**Affiliations:** 1 Department of Materials Science and Engineering, Beijing Key Laboratory of Failure, Corrosion and Protection of Oil/gas Facilities, China University of Petroleum (Beijing), Changping District, Fuxue Road 18, Beijing 102249, P. R. China; 2 State Key Laboratory of Solidification Processing, Northwestern Polytechnical University, Xi’an, Shaanxi 710072, China; 3 School of Mechanical and Chemical Engineering, the University of Western Australia, Crawley, WA 6009, Australia; 4 College of Science, China University of Petroleum (Beijing), Changping District, Fuxue Road 18, Beijing 102249, P. R. China

## Abstract

Mixing is an important method to improve the performance of surfactants due to their synergistic effect. The changes in bonding interaction and adsorption structure of IM and OP molecules before and after co-adsorbed on Fe(001) surface is calculated by DFTB+ method. It is found that mixture enable the inhibitor molecules with higher E_HOMO_ donate more electrons while the inhibitor molecules with lower E_LUMO_ accept more electrons, which strengthens the bonding interaction of both inhibitor agent and inhibitor additive with metal surface. Meanwhile, water molecules in the compact layer of double electric layer are repulsed and the charge transfer resistance during the corrosion process increases. Accordingly, the correlation between the frontier orbital (E_HOMO_ and E_LUMO_ of inhibitor molecules and the Fermi level of metal) and inhibition efficiency is determined. Finally, we propose a frontier orbital matching principle for the synergistic effect of inhibitors, which is verified by electrochemical experiments. This frontier orbital matching principle provides an effective quantum chemistry calculation method for the optimal selection of inhibitor mixture.

Surfactants play an important role in industrial production and daily life due to their unique interfacial activities^1^,^2^,^3^,^4^. A proper mixture of surfactants will produce synergistic effect, and thus exhibit better performance^5^,^6^,^7^. Until now, researchers have summarized some mixing principles for the mixture of surfactants according to various apparent parameters such as critical micelle concentration (CMC)^8^, hydrophobic lipophilic balance (HLB) value^9^, intermolecular interaction coefficientβ^10^,^11^, surface tension^12^, etc. From the microscopic perspective, these apparent parameters can influence or reflect the adsorption configuration and adsorption intensity of surfactant molecules at the interface^13^,^14^. Essentially, the changes of both molecular configuration and adsorption intensity are caused by the electron redistribution. As we all know that the frontier orbital theory (the highest occupied molecular orbital (HOMO) and the lowest unoccupied molecular orbital (LUMO)) of surfactants can be used to predict the electron transfer tendency when surfactant molecules adsorb on metal surface^15^,^16^. For the adsorption of inhibitor on metal surface, it has been shown that inhibitor molecules with higher E_HOMO_ are easier to donate electrons to metal. Similarly, the inhibitor molecules with lower E_LUMO_ are more inclined to accept electrons from metal^17^,^18^,^19^,^20^. However, the mixture of inhibitors involves the co-adsorption of various molecules on metal surface, and so far the problem how the adsorption interaction and the electrons transfer behavior during co-adsorption process has not been discussed, which limits the deep understanding of the synergistic mechanism of surfactant. From our viewpoint, the frontier orbital theory can also be used to analyze the change law for the electrons transfer of surfactants mixture, and then we can establish the correlation between the frontier orbital energy level and the synergistic effect of surfactants, which is helpful for understanding the synergistic mechanism of surfactants from the quantum mechanics perspective.

Currently, density functional based tight binding method (DFTB+) can realize quantitative calculation of electrons structure for a mixed system by semi-empirically calculating the wave function of extra-nuclear electron orbital and the interaction potential among atomic nucleus, which combines the accuracy of density functional theory (DFT) and the high efficiency of tight-binding method (TB)^21^,^22^,^23^,^24^. In this paper, imidazoline quantum salt (IM) and octyl phenol ethoxylates (OP) mixture system was selected as the studying object^25^. The changes of electron transfer and adsorption intensity of inhibitors before and after mixture were calculated using DFTB+ method. The correlation between the frontier orbital energy level and the inhibition efficiencies was analyzed. Finally, we propose a frontier orbital matching principle to explain the synergistic effect of inhibitor mixture and verify its validity via electrochemical experiment of 15 kinds of inhibitor mixtures. Therefore, the work of screening the mixed surfactant system according to large number of experiments can be simplified as screening and optimizing the mixed system according to the calculated molecular frontier orbital energy level distribution characteristics; meanwhile, this principle can be the guide for optimization and designation of the molecular structure of the surfactant.

## Results and Discussion

### The frontier orbitals of inhibitor molecules

**Figure 1:**
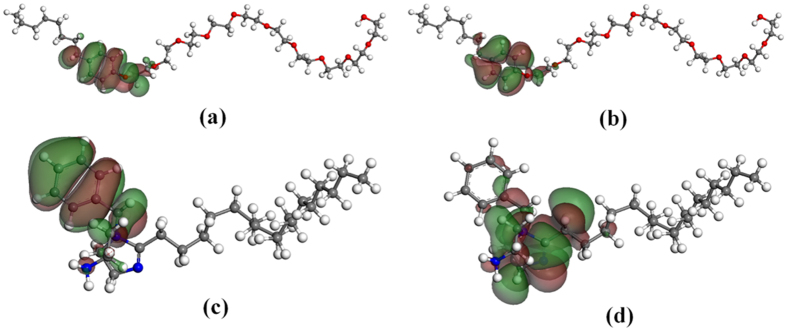
Distribution of HOMO orbital (**a**), LUMO orbital (**b**) of OP molecule, and HOMO orbital (**c**), LUMO orbital (**d**) of IM molecule.

Frontier orbitals of surfactant molecules usually locate at their active groups^26^. Figure 1 shows the distribution of HOMO and LUMO orbitals of OP and IM molecules, which is obtained by DMOL3 calculation. It is seen that the HOMO orbital of OP molecule locates at its benzene ring and the oxygen atom of polyoxyethylene chain attached to the benzene ring, while the LUMO orbital mainly locates at the benzene ring. This suggests that when OP molecule adsorbs on metal surface, the benzene ring of OP molecule can obtain and donate electrons while oxygen atom is mainly inclined to donate electrons. The HOMO orbital of IM molecule mainly locates at its benzene ring, while the LUMO orbital mainly locates at imidazoline ring. That means that the benzene ring of IM molecule can donate electrons whereas its imidazoline ring can obtain electrons. These active groups can adsorb on Fe surface through electron transfer.

### Electron transfer behavior of inhibitor molecules when adsorbs on Fe(001)

**Figure 2:**
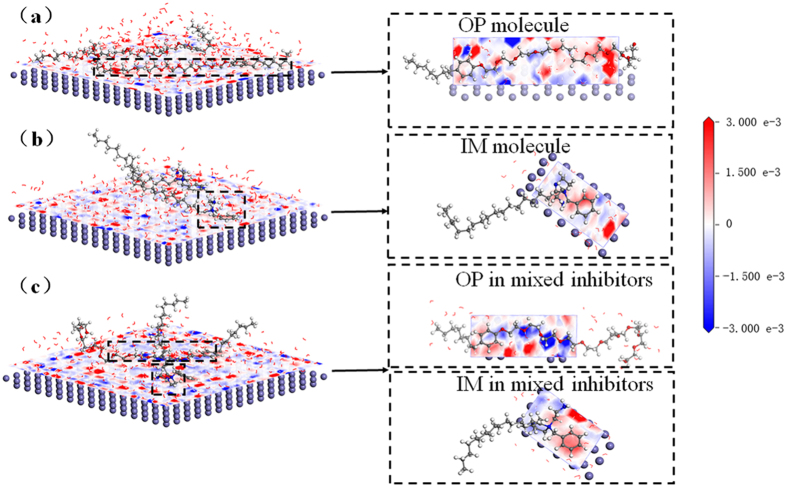
Adsorption configurations and electron density differences of OP (**a**), IM (**b**) and OP - IM mixtures (**c**) adsorption on Fe(001) surface. The planes between Fe(001) surface and inhibitor molecules are the cross-sections of electron density differences using Slide function in Material studio software. The red region shows an increase of electrons, while the blue area exhibits a lack of electrons. The deeper the color is, the larger degree it accepts or donates electrons.

Figure 2 shows the configurations and properties of OP molecules, IM molecules and OP-IM mixture after adsorbed on Fe(001) surface in aqueous environment through DFTB+ calculation. It is seen that when OP molecules adsorbs on Fe(001) surface, its benzene ring and polyoxyethylene chain adsorb on Fe surface in a parallel way, while the hydrophobic alkyl chain is away from Fe surface and stretches to aqueous solution. For IM molecules, the imidazoline ring and benzene ring adsorb on Fe surface and its hydrophobic alkyl chain is away from Fe surface and stretches to aqueous solution with a certain angle. When OP and IM molecules co-adsorb on Fe(001) surface as mixture, there is no change in the active adsorption sites of the two inhibitor molecules, but the number of adsorption sites in the polyoxyethylene chain of OP molecule decreases. Furthermore, the end of polyoxyethylene chain desorbs and stretches to aqueous solution, as shown in Fig. 2(c). The inclined angle of hydrophobic alkyl chain of IM molecule also increases. These results indicate that the adsorbed groups are the same as the active groups in the frontier orbital distribution calculation.

**Figure 3:**
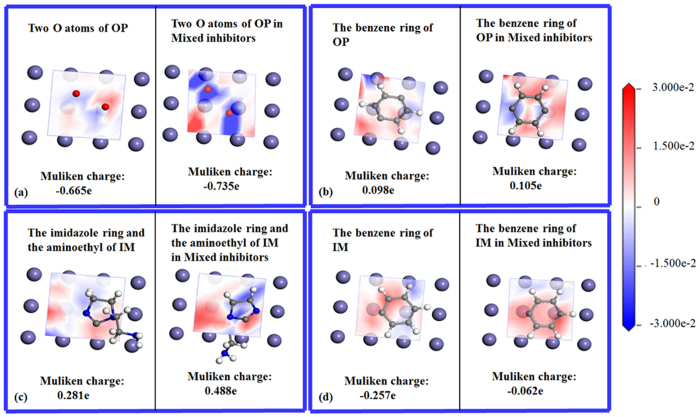
Electron density differences and Mulliken charges of the two O atoms in polyoxyethylene chain of OP molecule which is near benzene ring (**a**), benzene ring (**b**) of OP molecule, imidazoline ring and aminoethyl (**c**) and benzene (**d**) in IM molecule.

Figure 3 shows the electron density difference and Milliken charges of the active groups in OP and IM molecules before and after mixture. It is seen that when OP molecule adsorbs on Fe(001) surface, oxygen atoms in polyoxyethylene chain donate electrons obviously (Fig. 3(a)) whereas the benzene ring obtains a small amount of electrons (Fig. 3(b)). After mixture, oxygen atoms in polyoxyethylene chain of OP molecule lose more electrons, while there is no significant change in the amount of electrons of benzene ring. In other words, OP molecule donates more electrons after mixing with IM.

Similarly, when IM molecule adsorbs on Fe(001) surface, imidazole ring and aminoethyl group obtain electrons obviously (Fig. 3(c)) whereas the benzene ring donates electrons (Fig. 3(d)). After mixture, imidazole ring obtains more electrons and benzene ring also obtains electrons, as shown in Fig. 3(c,d). This indicates that IM molecule obtains more electrons after mixing with OP. The feature of active groups in OP and IM molecules to donate or accept electrons is in good agreement with their trend to donate or accept electrons presented in the frontier orbitals distribution.

**Figure 4:**
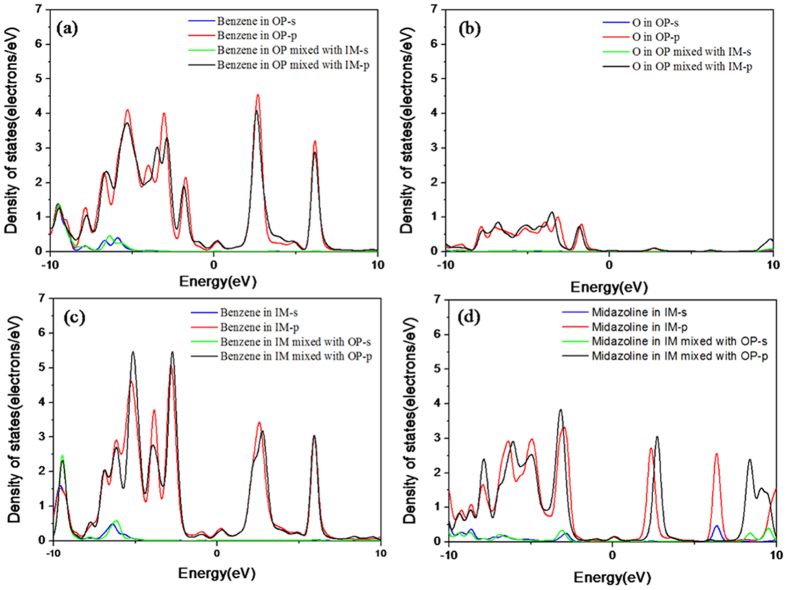
PDOS of benzene ring (**a**) and O atoms (**b**) of OP molecule before and after mixing with IM molecule, and the PDOS of the imidazoline ring (**c**) and the benzene ring (**d**) of IM molecule before and after mixing with OP molecule.

The electron transfer behavior will affect the adsorption intensity of inhibitor molecules on metal surface. Figure 4 shows the partial density of states (PDOS) of the active groups in OP and IM molecules before and after mixture. The corresponding adsorption intensities of the active groups in inhibitor molecules were analyzed. It is seen that after mixture, DOS peaks of s and p orbitals of the benzene ring and oxygen atom in OP molecule, and the imidazoline ring in IM molecule shift to lower energy, the peak position of the benzene ring in IM are hardly changed. This indicates that both the adsorption of OP and IM molecules on Fe surface enhance after mixture.

As we all know, some groups will be defined as gauche if they have a synclinal alignment of groups attached to adjacent atoms^27^. After the mixing of OP molecule and IM molecule, the angle between the imidazoline ring of IM molecule and metal surface increased (as shown in the Supplementary information, Fig. 1(a)), and the C-C adjacent to the imidazoline ring maintain the staggered conformation. This may lead to an increase of inclined angle between hydrophobic alkyl chain of IM molecule and metal surface. Since the adsorption interaction of O atoms in the middle part of polyoxyethylene chain of OP molecule on metal surface is stronger, the number of adsorption sites in the polyoxyethylene chain of OP molecule decreases, and the end-piece of polyoxyethylene chain desorbed from metal surface (as shown in the Supplementary information, Fig. 1(b)). According to previous literatures^28^,^29^,^30^, the C-C bond in O-C-C-O bond sequence has a gauche preference due to its first-order σ interaction. Therefore, the end-piece of polyoxyethylene chain stretches to aqueous solution. So the reason for the configuration change of inhibitor molecules before and after mixing has been explained.

### Inhibition efficiency of IM-OP mixture

The corrosion rates of L245 steel were measured by linear polarization method in 3% NaCl solution without inhibitor and with 100 ppm OP, 100 ppm IM or 20 ppm OP + 80 ppm IM mixture (see in Table 1). The corrosion rates of L245 steel in these four conditions were 5.098 mm/a, 0.831 mm/a, 0.500 mm/a and 0.377 mm/a, respectively. Compared to IM inhibitor, the inhibition efficiency of OP-IM mixture increases by 24.6%, indicating obvious synergistic effect between IM and OP.

For the inhibition of inhibitors to the corrosion of metals, on one hand, inhibitors will form an adsorbed film on metal surface to hinder corrosive species to metal surface, and thus the corrosion of metals will be inhibited^31^,^32^,^33^. On the other hand, the adsorption of inhibitor molecules will increase the distance of the compact layer of double electric layer from metal surface. Then the charge transfer resistance will increase and thus the electrochemical corrosion process is inhibited^34^,^35^.

**Figure 5:**
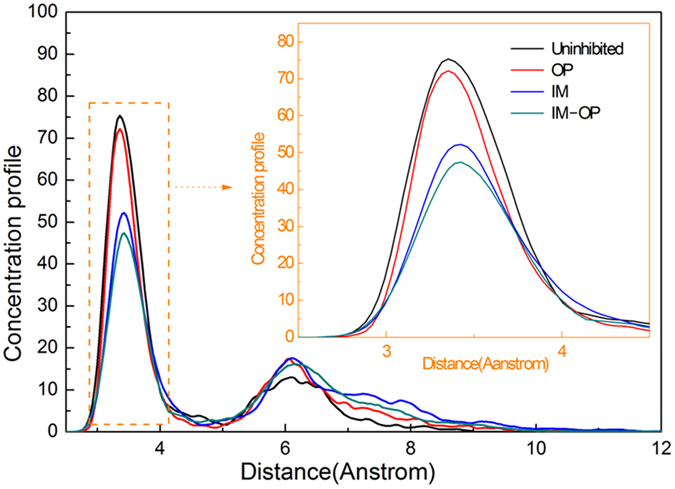
The quantity distribution of water molecules and the distance of water concentration peaks to the metal surface.

Figure 5 shows the distribution of water molecules along the distance from metal surface. It is seen that in blank solution (only water molecules is present), the peak of water molecule amount appears at the position about 3.33 Å from Fe surface. When OP or IM molecules are added into solution, the peak of water molecule amount appears at 3.36 Å and 3.42 Å, respectively. Furthermore, the amount of water molecules in the compact layer decreases obviously. When IM and OP molecules are present simultaneously, water molecules move further away from metal surface and the distance increases to 3.44 Å. The amount of water molecules in compact layer also further decreases. This situation indicates that the amount of water molecules in compact layer will decrease and compact layer will move away from metal surface as the adsorption interaction of OP and IM molecules on Fe surface becomes stronger.

### Relationship between the frontier orbital energy level, the electrons transfer and the synergistic effect of inhibitors

**Figure 6:**
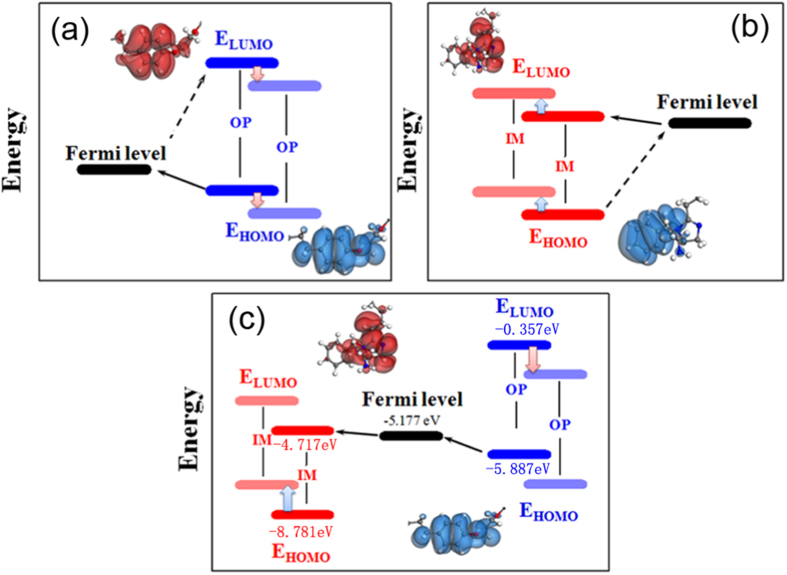
Frontier orbital energetic positions of OP molecule (**a**), IM molecule (**b**) and IM + OP molecules (**c**) adsorption on Fe(001) surface.

The bonding interaction between adsorbed molecules and metal surface depends on the frontier orbital energetic position of adsorbed molecules and the Fermi level of metal^36^. Figure 6 shows the frontier orbital energies of IM and OP molecules before and after mixture. For OP molecule, the E_HOMO_ (−5.887 eV) of OP molecule is close to the Fermi level of Fe(−5.177 eV) while the E_LUMO_ (−0.305 eV) of OP molecule is far away from the Fermi level of Fe. Thus, electrons likely excited from the HOMO orbital of OP molecule to Fe surface. Moreover, the amount of the transferred electrons is large. However, the energy gap between E_LUMO_ of OP molecule and the Fermi level of Fe is large. Therefore, it is difficult for the electrons to be excited from Fe surface to the LUMO orbital of OP molecule. Generally speaking, OP molecule donates electrons and thus electrons accumulate on Fe surface until the electron transfer reaches equilibrium. During this process, the frontier orbital energetic positions of OP molecule shift to the lower level, while the Fermi level of Fe is considered to be no significant change^37^.

For IM molecule, the E_LUMO_ (−4.717 eV) of IM molecule is close to the Fermi level of Fe while its E_HOMO_ (−8.781 eV) is far away the Fermi level of Fe. Under this situation, it is hard for electrons to be excited from the HOMO orbital of IM molecule to Fe surface. However, the electrons are likely excited from Fe surface to the LUMO orbital of IM molecule. Therefore, IM molecule accepts electrons and positive charges accumulate on Fe surface until the electron transfer reaches equilibrium. During this process, the frontier orbital energetic positions of IM molecule shift to higher level, while there is insignificant change in the Fermi level of Fe.

For IM-OP mixture, the E_LUMO_ (−4.717 eV) of IM molecule and the E_HOMO_ (−5.887 eV) of OP molecule are close to the Fermi level of Fe(−5.177 eV). Therefore, the electrons are likely to transfer from the HOMO orbital of OP molecule to Fe surface, and then transfer from Fe surface to the LUMO orbital of IM molecule. Under this situation, as the electrons transfer from Fe surface to IM molecule, the accumulated electrons on Fe surface decreases, which is beneficial to the further electron transfer from the HOMO orbital of OP molecule to Fe surface. Similarly, as electrons transfer from OP molecule to Fe surface, the amount of electrons on Fe surface increases, which is helpful for the further electron transfer from Fe surface to IM molecule. Accordingly, OP molecule can donate more electrons while IM molecule can accept more electrons when they co-adsorb on Fe surface, which results in stronger bonding interaction of IM and OP molecules and Fe surface.

Generally speaking, according to the aforementioned results, compared with IM or OP adsorbed on Fe(001) surface, when OP and IM molecules co-adsorb on Fe(001) surface, as the Fermi level of Fe is close to the E_LUMO_ of IM molecule and the E_HOMO_ of OP molecule, more electrons are likely to transfer from the HOMO orbital of OP molecule to Fe surface, and more electrons are likely to transfer from Fe surface to the LUMO orbital of IM molecule. Then the bonding intensity of both OP and IM molecules with Fe surface increases. Therefore, it will not only beneficial for the exclusion of water molecules in the compact layer away from Fe surface, but also helpful for the decrease of the number of water molecules in the compact layer. Therefore, the charge-transfer resistance in the electrochemical corrosion will increase, and the corrosion rate of metal will decrease significantly. Thus the inhibitors mixture shows a synergistic effect.

Based on the aforementioned insights, we further consider that when inhibitor molecules with higher E_HOMO_ (higher or closed to the Fermi level of Fe) mix with inhibitor molecules with lower E_LUMO_ (lower or closed to the Fermi level of Fe), the inhibitor molecules with higher E_HOMO_ is likely to donate more electrons to metal surface while the inhibitor molecules with lower E_LUMO_ is likely to accept more electrons to metal surface. Therefore, the adsorption intensity of inhibitor molecules will be further strengthened. That is to say, we can judge the synergistic effect of inhibitors mixture through the frontier orbital energies of inhibitor molecules and the corresponding Fermi level of metal. Accordingly, we can optimize the component of inhibitors mixture, which is called “the frontier orbitals matching principle” for synergistic effect.

### Experimental verification for the frontier orbitals matching principle

In order to verify the frontier orbitals matching principle for synergistic effect, three kinds of cationic surfactants (IM, KL and BD) were chosen as inhibitors, and two kinds of anionic surfactants (SLS and SDS) and three kinds of nonionic surfactants (OP, FE and BTAH) were used as additives. The electron transfer tendencies of these molecules were determined according to the frontier orbital energetic positions, and then the inhibition efficiency tendency of inhibitors mixture was predicted. The inhibition efficiency of inhibitors mixture was determined by using electrochemical linear polarization method to verify the frontier orbitals matching principle for synergistic effect.

**Figure 7:**
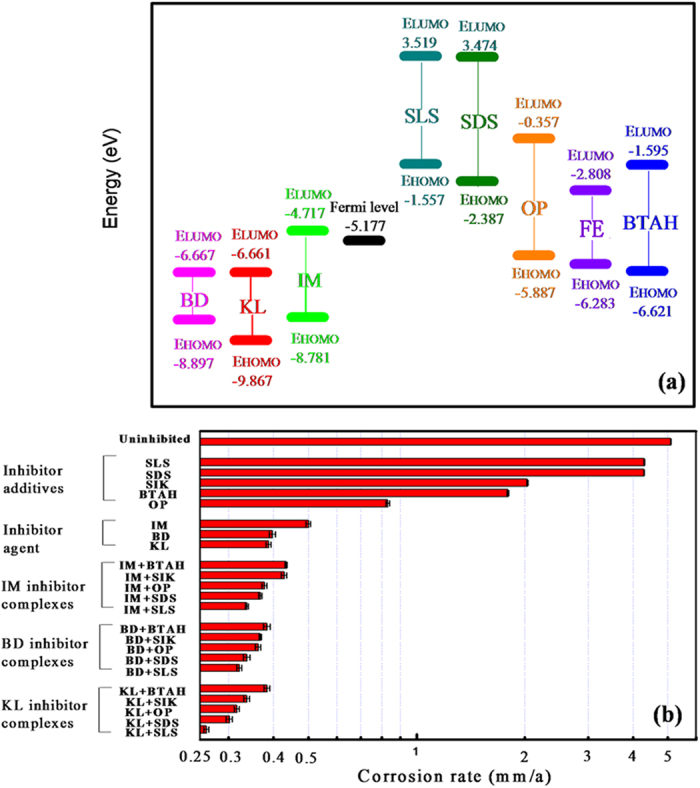
Frontier orbital energetic positions of surfactants (**a**) and the inhibition efficiency of inhibitors (**b**).

The frontier orbital energy positions of the selected 8 kinds of surfactants are shown in Fig. 7(a). It is seen that the E_LUMO_ of both KL and BD is lower than those of others. This means that these two kinds of molecules present the best ability in accepting electrons, while SLS and SDS show the best tendency in donating electrons. Therefore, we predict that the mixed systems of these surfactants would form strong adsorption interactions with metal surface, and then present high inhibition efficiencies. However, the electron donating ability of BTAH and the electron accepting ability of IM is the weakest, so the inhibition efficiency of IM-BTAH mixture will be low.

Figure 7(b) shows the inhibition efficiencies of various mixed systems according to the electrochemical linear polarization. It’s obvious that the inhibition efficiencies of the 5 inhibitor additives are low (for example, 15.6% for SLS and 15.8% for SDS, respectively). The inhibition efficiencies of the three selected inhibitor agent (IM, KL and BD) are higher. However, the inhibition efficiencies of the mixture of them obviously increase. Among them, the inhibition efficiency of KL + OP shows the highest inhibition efficiency while IM + BTAH complex presents the lowest inhibition efficiency. The electrochemical measurements are in accordance with the prediction according to the frontier orbitals matching principle.

It is noteworthy that although the E_LUMO_ of BD molecule is a little lower than that of KL molecule, after mixed with inhibitor additives, BD mixture systems show lower inhibition efficiency than those of KL mixture systems. This situation is probably attributed to the small difference between the E_HOMO_ and E_LUMO_ of BD molecule. Then, electrons are easy to be excited from the HOMO orbital to the LUMO orbital of BD molecule. Therefore, the electron accepting process of the LUMO orbital of BD molecule from Fe surface is affected, and the bonding interaction of BD molecule and Fe surface is weakened.

In summary, the electron transfer behavior of the co-adsorbed IM and OP molecules on Fe(001) surface under water environment was calculated using DFTB+ method. The electron transfer tendency was the same as the tendency predicted through frontier orbital energy. Thus the enhanced inhibition efficiency of inhibitors mixture is explained with the quantum mechanics. Furthermore, the optimal selection for the component of inhibitors mixture system is proposed based on the frontier orbitals matching principle. The mixture of inhibitor molecules with higher E_HOMO_ and the other with lower E_LUMO_ will show higher inhibition efficiency. The inhibition efficiencies of 15 kinds of mixed inhibitor systems, which are obtained using electrochemical method, show the same tendency as that predicted by the frontier orbitals matching principle. Thus a simple method is provided for the optimal selection of the component in the mixed inhibitors system. Using this principle we can optimize some potential mixed inhibitors systems according to the calculated molecular frontier orbital energy level distribution instead of so large number of screening experiments when the number of surfactants molecules available for selection is very large. So this principle also has the same important guiding significance in the mixture of surfactants.

## Conclusions

The changes of the bonding interactions and the adsorption structures of mixed IM and OP molecules on Fe(001) surface was calculated using DFTB+ method. It shows that the inhibitor molecule with higher E_HOMO_ donates more electrons while the inhibitor molecule with lower E_LUMO_ accepts more electrons after mixture. Therefore, both the bonding interactions of inhibitors and inhibitor additives with metal surface are strengthened, and water molecules in the compact layer are further excluded. Moreover, the charge transfer resistance during the corrosion process increases. Accordingly, the frontier orbitals matching principle for the synergistic effect is proposed. The relationship between the E_HOMO_ and E_LUMO_ of the molecules in the mixed system, the Fermi level of metal and the inhibition efficiency of inhibitors mixture is established. The proposed principle is also verified using electrochemical method. Thus a simple quantum mechanics method for the optimal selection of the component in the mixed inhibitor system and the design of the surfactants is provided.

## Materials and Methods

### Modeling and theoretical methods

Firstly, interface structures of the inhibitors adsorbed on Fe(001) surface in aqueous environment were built. Then molecular dynamics simulation was carried out to obtain the most stable configuration as the initial interface structure for DFTB+ calculation. Subsequently, DFTB+ method was used to calculate the electronic structures and properties of interface structure, such as charge difference density, Milliken charge and partial density of states, to study the adsorption and electrons transfer behavior of inhibitor molecules on Fe(001) surface. Theoretical computations mentioned above were performed using Materials Studio (Accelarys Inc.) package^38^. Finally, the frontier orbital of inhibitor molecules were determined by means of Gaussian 09 software package^39^.

### Modeling

The interface structures with periodic boundary conditions were composed of Fe surface and solution containing water molecules and inhibitor molecules. Firstly, a metal surface, consisting of four layers, was built. Its size was 43.0 Å × 43.0 Å × 1.4 Å, containing 450 Fe atoms. All the atoms on metal surface were fixed during simulation process. Secondly, the solution layers were built, containing 1500 water molecules and 2 inhibitor molecules (referring to 2 OP molecules, 2 IM, or 1 OP molecule and 1 IM molecule for complex inhibitor). Finally, the interface structures were built by combining Fe surface with solution layers, and a vacuum region of 15.0 Å was set to avoid any arbitrary boundary effects^40^.

### Molecular dynamic (MD) calculation

Firstly, water/inhibitors/Fe(001) system were optimized to minimum energy using smart minimize tools using COMPASS force field^41^,^42^,^43^. Then MD simulation was performed using the Andersen thermostat^44^ at 298 K, NVT emsemble, with a time step of 1fs and the total simulation time of 100 ps. Finally, the lowest energy structure of inhibitor molecules adsorbed on Fe(001) in water environment were extracted from the balanced structures, which can be used as the initial interface structure for DFTB+ calculation.

### Density functional based tight binding (DFTB+) calculation

The interface structures obtained through molecular dynamic calculation were further optimized to minimum energy using DFTB+ method, and then the electronic structures and properties were calculated, including charge difference density, Milliken charge and partial density of states. In order to reduce the calculation cost, only 308 water molecules near metal surface were considered. During all the DFTB+ calculation, the Trans3d was used to describe the interaction of atoms^45^,^46^ and dispersion interactions^47^,^48^,^49^ were considered. The convergence criteria are listed as follows: energy is 0.02 Kcal/mol; force is 0.1 Kcal/mol/Å; displacement is 0.001 Å; SCC tolerance is 10^−8^ and K-point mesh is 1 × 1 × 1.

### Density functional theory calculation of frontier orbitals

The frontier orbital distributions of inhibitors were calculated using the Gaussian 09 software programs^39^ by means of the functional HSEh1PBE density functional theory formalism (DFT) and the 6–31G orbital basis set for all atoms. Two main quantum related parameters were considered: the energy of the highest occupied molecular orbital (E_HOMO_) and the energy of the lowest unoccupied molecular orbital (E_LUMO_).

### Materials

Material used as working electrode was L245 mild steel, containing 0.13 wt.% C, 0.76 wt.% Mn, 0.014 wt.% S, 0.022 wt.% P, 0.24 wt.% Si, and iron balance. Test solution was 3 wt.% NaCl solution, and the pH was adjusted to 3.0 using analytical grade sulfuric acid. Three kinds of inhibitors (imidazoline quantum salt (IM), quinoline quaternary ammonium salt (KL) and hexadecylpyridinium chloride pyridine (BD)) and five kinds of additives (Sodium dodecylbenzenesulfonate (SLS), Sodium dodecyl sulfate (SDS), octyl phenol ethoxylates (OP), fluorinated ether compound (FE) and benzotriazol (BTAH)) were used (chemical structure are shown in Supplementary Scheme 1). Among them, IM and KL were synthesized according to references^50^,^51^, and the productivities of IM and KL were 91.2% and 90.5%, respectively. Meanwhile, the structure of IM and KL were verified by infrared spectrum (IR). The other reagents were analytical grade. All the inhibitor mixtures involved were composed of 80 wt. % inhibitor and 20 wt. % additive.

### Electrochemical measurements

Electrochemical measurements were carried out in a conventional three-electrode cell, using a Solartron-SI 1280B electrochemical workstation. L245 steel was used as working electrode. Graphite rod was used as counter electrode. Saturated calomel electrode (SCE) with Luggin capillary salt bridge was used as reference electrode. Specimens with size of ø12 × 5 mm were cut from L245 steel as working electrode. Prior to each experiment, the working electrode was mechanically abraded with emery paper from 400 # to 2000 #, then polished with diamond abrasive (0.5 μm) to a mirror face. The electrode was rinsed with double-distilled water and dried in air.

Before each test, the test solution was purged with N_2_ (99.999%) for 2 h, and the open circuit potential (OCP) was recorded at the same time. Then 100 ppm IM or 100 ppm IM + OP was added in the test solution, respectively. CO_2_ (99.999%) was introduced into the test solution for 30 min. Finally, linear polarization measurements were performed by polarization from −10 mV ~ 10 mV vs. OCP with a scan rate of 0.1 mV·s^−1^. All potentials reported here are referred to SCE.

## Additional Information

**How to cite this article**: Han, P. *et al.* Study on Synergistic Mechanism of Inhibitor Mixture Based on Electron Transfer Behavior. *Sci. Rep.* **6**, 33252; doi: 10.1038/srep33252 (2016).

## Supplementary Material

Supplementary Information

## Figures and Tables

**Table 1:** Corrosion rates of L245 steel in 3 wt.% NaCl solution, 3 wt.% NaCl solution with 100 ppm OP, 3 wt.% NaCl solution with 100 ppm IM and 3 wt.% NaCl solution with 20 ppm OP + 80 ppm IM.

Inhibitor	Corrosion rate (mm/a)
no inhibitor	5.098
OP	0.831
IM	0.500
IM + OP	0.377
