# Supplementary material for: Study on Synergistic Mechanism of Inhibitor Mixture Based on Electron Transfer Behavior
Source: Sci Rep. 2016 Sep 27;6:33252. doi: 10.1038/srep33252 (PMC5037402; doi:10.1038/srep33252)
Supplement: Supplementary Information [file srep33252-s1.doc]

supplementary information

Study on Synergistic Mechanism of Inhibitor Mixture Based on Electron Transfer Behavior

Peng Han^1, #^, Yang He^1, #^, Changfeng Chen^1^,*, Haobo Yu^1^,*, Feng Liu^2^,*, Hong Yang^3^ , Yue Ma^4^ & Yanjun Zheng^1^

1. Department of Materials Science and Engineering, Beijing Key Laboratory of Failure, Corrosion and Protection of Oil/gas Facilities, China University of Petroleum (Beijing), Changping District, Fuxue Road 18, Beijing 102249,P. R. China
2. State Key Laboratory of Solidification Processing, Northwestern Polytechnical University, Xi'an, Shaanxi 710072, China
3. School of Mechanical and Chemical Engineering, the University of Western Australia, Crawley, WA 6009, Australia
4. College of Science, China University of Petroleum (Beijing), Changping District, Fuxue Road 18, Beijing 102249,P. R. China

#These authors are co-first authors.

*These authors are corresponding authors. Correspondence to Changfeng Chen ([Chen_c_f@163.com](mailto:Chen_c_f@163.com)), Haobo Yu([Yu_h_b@sina.com](mailto:Yu_h_b@sina.com)) or Feng Liu ([liufeng@nwpu.edu.cn](mailto:liufeng@nwpu.edu.cn)).

IM KL

BD

SLS SDS

OP FE

BTAH

Supplementary Scheme 1 Chemical structures of the inhibitors and additives


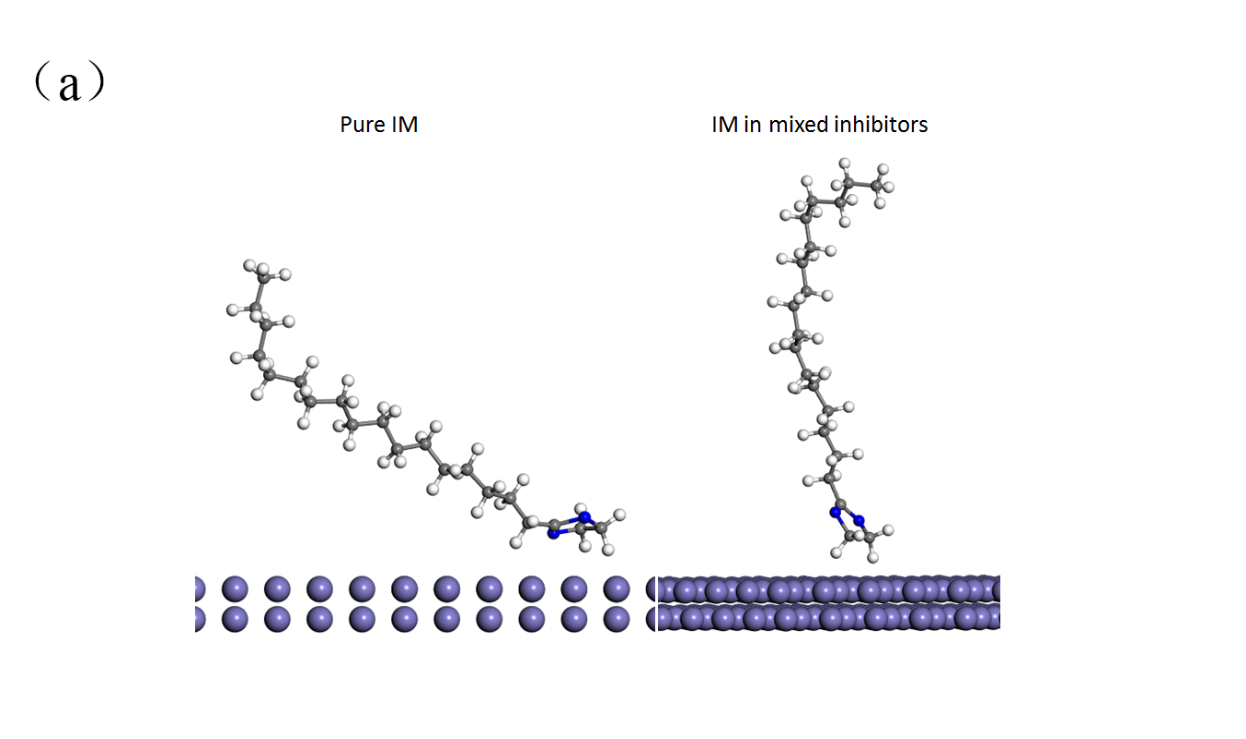

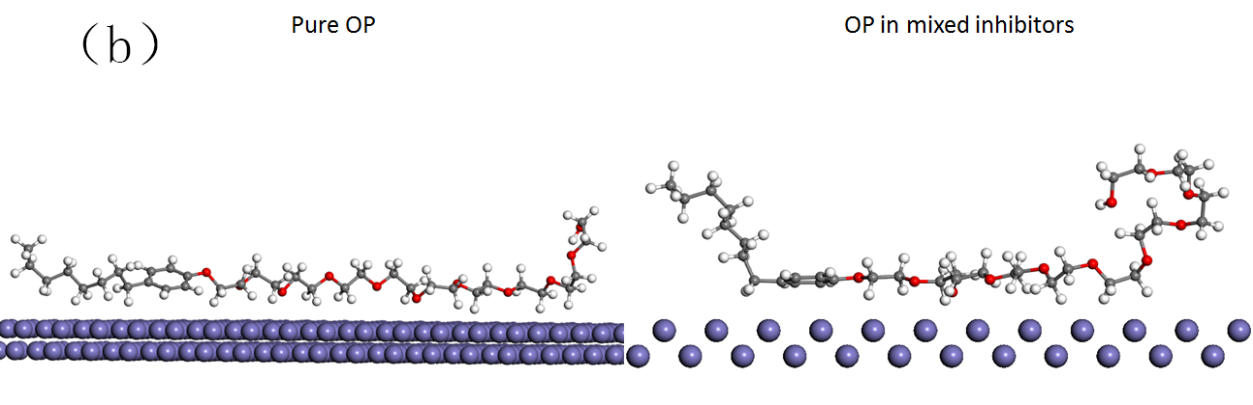


Supplementary Figure 1 Adsorption configurations of the inhibitors and additives (a) Imidazole ring and the hydrophobic alkyl chain in IM molecules in pure IM system and in mixed inhibitors system (Note: In order to give a better understanding of the adsorption configurations, some of the atoms are not displayed in the figure); (b) OP molecules in pure OP system and in mixed inhibitors system.
